# Optimization of the Use of His_6_-OPH-Based Enzymatic Biocatalysts for the Destruction of Chlorpyrifos in Soil

**DOI:** 10.3390/ijerph14121438

**Published:** 2017-11-23

**Authors:** Olga Senko, Olga Maslova, Elena Efremenko

**Affiliations:** Faculty of Chemistry, Lomonosov Moscow State University, Moscow 119991, Russia; senkoov@gmail.com (O.S.); olga.maslova.rabota@gmail.com (O.M.)

**Keywords:** hexahistidine-containing organophosphorus hydrolase, soil, chlorpyrifos biodegradation, Dursban, agricultural pesticides, organophosphorus and organochlorine compounds

## Abstract

Applying enzymatic biocatalysts based on hexahistidine-containing organophosphorus hydrolase (His_6_-OPH) is suggested for the decomposition of chlorpyrifos, which is actively used in agriculture in many countries. The application conditions were optimized and the following techniques was suggested to ensure the highest efficiency of the enzyme: first, the soil is alkalinized with hydrated calcitic lime Ca(OH)_2_, then the enzyme is introduced into the soil at a concentration of 1000 U/kg soil. Non-equilibrium low temperature plasma (NELTP)-modified zeolite is used for immobilization of the relatively inexpensive polyelectrolyte complexes containing the enzyme His_6_-OPH and a polyanionic polymer: poly-l-glutamic acid (PLE_50_) or poly-l-aspartic acid (PLD_50_). The soil’s humidity is then increased up to 60–80%, the top layer (10–30 cm) of soil is thoroughly stirred, and then exposed for 48–72 h. The suggested approach ensures 100% destruction of the pesticide within 72 h in soils containing as much as 100 mg/kg of chlorpyrifos. It was concluded that using this type of His_6_-OPH-based enzyme chemical can be the best approach for soils with relatively low humus concentrations, such as sandy and loam-sandy chestnut soils, as well as types of soil with increased alkalinity (pH 8.0–8.4). Such soils are often encountered in desert, desert-steppe, foothills, and subtropical regions where chlorpyrifos is actively used.

## 1. Introduction

The ever-continuing growth of the Earth’s population is accompanied with an increasing demand for agricultural products: fruit, vegetables, cereals, meat, etc. Strategic development programs in many countries address the problem of stability and security of food supply and therefore support improvement of agricultural practices [1,2]. Development of animal husbandry and plant cultivation implies an increase of the areas used for grazing and hay harvesting as well as expansion of arable land and quick deforestation of territories [3,4,5]. However, since most areas with humus-rich soils are already being used in agricultural processes, such development calls for increasing use of steppes and deserts, which heretofore have been largely usused. This, in turn requires optimization of plant cultivation practices on relatively poor soils in terms of organic and mineral composition, such as sandy and loam-sandy chestnut soils [6].

The trend towards higher economical efficiency has led to the widespread utilization of organophosphorus and organochlorine agricultural pesticides as an inevitable part of modern agricultural practices. The application of pesticides against various plant pests is currently well founded from the economical viewpoint [7,8,9,10]. Chlorpyrifos in the form of the commercial chemical Dursban is a broad-spectrum chlorinated organophosphate insecticide, nematicide, and acaricide. The chlorpyrifos demand has increased annually [11] due to this pesticide’s high efficiency and low market price. Chlorpyrifos-based chemicals are widely used in agriculture in many countries [6,7,11,12], oftentimes for cultivating palms and other plants in subtropical regions on soils with poor humus content [13,14].

The toxicity of chlorpyrifos is rather high compared to that of other organophosphate pesticides, and so is its half-life, which can be (depending on many factors) as long as 120 days [11]. The non-decomposed residual chlorpyrifos presents a great hazard [15], because this chemical affects the soil biocenosis inhabitants [16,17,18]. The residual pesticide is leached from the soil and ends up in water bodies; besides, in can be carried via forage into the organisms of agricultural animals [19] and also get into humans via the food chain or by other means, which has a negative impact on human health [20,21]. One of the most common human exposure pathways to the pesticide is directly through pesticide-contaminated surface soil. Humans can be thus exposed to pesticides by ingesting soil, inhaling soil dust, and through dermal contact [20], especially for children [21].

The gravest health consequences are registered for agricultural workers and the general populace in the vicinity of pesticide application sites [11,19,22,23,24]. The removal of residual chlorpyrifos from the soil is one of the possible solutions for preventing the negative impact of this chemical on humans and animals. Thus, the search for efficient techniques for destroying the non-decomposed chlorpyrifos residues in various media (including soil) is a topical problem for researchers [11,25,26,27,28].

The existing techniques for purification of pesticide-polluted soils can be classified as in-situ (on-site purification of soils) and ex-situ ones (soil removal, purification at remote facilities, followed by returning of the soil to the original location) [29]. The former technique is easier to realize. The second classification type is further divided according to the method involved into non-biological (chemical, thermal, or physico-chemical) of biological ones (or their combination). However, the modern requirements for environmental safety make introduction into the soil of other active substances, e.g., strong oxidizers, an inacceptable solution, therefore biological soil purification techniques are preferred [30]. The toxicity of pesticide-contaminated soils can be reduced by remediation processes involving the use of microbes (bioremediation) [31,32,33], plants (phytoremediation) [34,35], plant-microorganism associations and extracellular and/or cell-free enzymes. Cells degrade or use the pesticides by various co-metabolic processes [36,37]. Many microorganisms and other soil inhabitants can efficiently decompose chlorpyrifos [11]. Soil bioremediation techniques involving the use of live organisms have a number of essential limitations. Bioremediation of soils with relatively poor humus content is especially difficult because the live cells used in the process lack sustenance. However, this type of soils is becoming increasingly involved in the agricultural process [38,39]. The use of extracellular and/or cell-free enzymes has been proposed as a remediation technique to purify such soils. The best results are achieved when using enzymes in immobilized form [40,41,42,43]. The immobilized enzymes have higher stability, they help ensuring high biocatalyst concentration in the immediate vicinity of the pollutant, which facilitates destruction of the latter. The enzymes are highly selective in their activity, and the corresponding chemicals are easy to standardize and store. The enzymes are more mobile compared to microorganism cells, and can preserve their activity in the presence of both high and low concentration of toxic substances [44].

The enzymatic biocatalysts based on hexahistidine-containing organophosphorus hydrolase (His_6_-OPH) are very useful for the elimination of organophosphorous compounds (OPCs) accumulating in soils treated with pesticides, since the enzyme has a wide enough range of activity and is capable of hydrolyzing various OPCs and even their destruction products [43,45,46].

Since the pH optimum of His_6_-OPH action (10.5) [47] is significantly higher than the neutral value, which is typical for most soils, some additional stabilization of the enzyme introduced into the soil should be undertaken. The stabilization of the activity of His_6_-OPH enzyme can be achieved by sorption immobilization on a carrier, but also by the formation of the enzymatic polyelectrolyte complexes (EPCs) [45,48,49]. Such EPCs can be produced by simple mixing of the enzyme and polymer solutions that act as oppositely charged ion exchangers, and the optimum concentrations of the respective substances are to be found experimentally.

The search for efficient soil purification techniques has led to the choice of poly-l-glutamic acid (PLE_50_) and poly-l-aspartic acid (PLD_50_) as polyanions for the formation of complexes with His_6_-OPH. These preferences for certain complexes were based on the results of the previously performed computer design of EPCs [50]. The use of such amino acid polymers as the enzyme partners within the EPC appeared attractive because in the case of biodegradation of such complexes under ambient conditions, the release of amino acid residues should occur that could serve as digestible nitrogen sources for the soil microbiota, thus positively stimulating the distribution of indigenous microorganisms. The results of preliminary studies indicated the excellent perspectives of using His_6_-OPH-based biocatalysts for bioremediation of soils polluted with paraoxon [43].

Thus, the purpose of this work was to develop a strategy for Dursban (chlorpyrifos) detoxification based on the use of relatively inexpensive polyelectrolyte complexes containing the enzyme His_6_-OPH and the polyanionic polymers poly-l-glutamic acid (PLE_50_) or poly-l-aspartic acid (PLD_50_), in various types of soil. The His_6_-OPH and its EPCs were used both in free and immobilized form. Immobilization was achieved via sorption in natural zeolite with improved sorption capacity owing to its modification by treatment with non-equilibrium low temperature plasma (NELTP) in a barrier discharge.

## 2. Materials and Methods

### 2.1. Materials

Natural zeolite (1.6 ÷ 4.3 mm fraction) (ZAO Orel Zeolite, Oryol, Russia) was used as carrier for His_6_-OPH immobilization. Poly-l-glutamic acid sodium salt (PLE_50_, molecular weight (MW) = 7500 Da) and poly-l-aspartic acid sodium salt (PLD_50_, MW = 6800 Da) (Alamanda Polymers, Huntsville, AL, USA) were used for forming the polyelectrolyte complexes of His_6_-OPH enzyme. We also used hydrated calcitic lime, Ca(OH)_2_ provided by “Ilishevskaya Selkhozkhimiya” MUE (Verkhneyarkeyevo, Bashkortostan, Russia), dolomite powder with total mass fraction of calcium and magnesium carbonates (CaCO_3_ + MgCO_3_) 80% provided by Gera LLC (Lytkarino, Russia) and birch ash provided by Vitaflor LLC (Krasnogorsk, Russia). Dursban 480 EC containing 480 g of chlorpyrifos (*O*, *O*-diethyl O-3, 5, 6-trichloro-2-pyridinyl phosphorothioate, CPY; Chemical Abstract Service number (CAS No.) 2921-88-2) per 1 L was produced by Dow AgroSiences LLC (Indianapolis, IN, USA), paraoxon and other reagents were purchased from Sigma (St. Louis, MO, USA). Soil samples were provided by the Soil Science Department of the Lomonosov Moscow State University (sand: pH 7.4 ± 0.2%, humidity 90 ± 5%, humus 0.0 ± 0.2%; grey forest: pH 6.5 ± 0.3%, humidity 71 ± 4.5%, humus 1 ± 0.3%; chestnut: pH 8.0 ± 0.2%, humidity 82 ± 6%, humus 2.1 ± 0.1%; chernozem pH 7.0 ± 0.2% humidity 75 ± 5%, humus 6.7 ± 0.4%).

### 2.2. Preparation for Analysis of Enzyme Samples and Enzyme Polyelectrolyte Complexes

Recombinant *Escherichia coli* strain SG13009[pREP4] (Qiagen, Hilden, Germany) transformed by plasmid encoding His_6_-OPH [51] was used for enzyme production. Cells were cultivated, and the enzyme was isolated and purified as described previously [47,52]. 

Cell biomass (20 g) was suspended in 50 mM phosphate buffer (pH 8.0) (200 mL) containing 0.3 M NaCl and homogenized by sonication on ice. Cell debris was removed by centrifugation for 30 min at 14,000× *g*. An equal volume of Ni-NTA agarose pre-equilibrated in 50 mM phosphate buffer was added to the supernatant. The resulting suspension was packed in a chromatographic column (5 mL) and washed with 50 mM phosphate buffer (pH 8.0) containing 300 mM NaCl and 10 mM imidazole at the flow rate of 0.5 mL/min until the absorbance (λ = 280 nm) decreased to 0.01. The enzyme was eluted by an imidazole concentration gradient (10–500 mM). Imidazole was removed from collected protein fractions by dialysis against 50 mM phosphate buffer, pH 8.0, and after that the fractions were analyzed, and protein concentration and enzymatic activity were determined.

The concentration of protein was determined by Bradford assay. The level of His_6_-OPH expression as well as homogeneity of enzyme preparations was estimated by electrophoresis under denaturing conditions in 12% polyacrylamide gel using a Miniprotean II cell (Bio-Rad, Hercules, CA, USA) followed by Coomassie Blue (R-250) staining. According to SDS-PAGE data, the homogeneity of His_6_-OPH was similar to that obtained previously (98%) [52].

The purified preparation of His_6_-OPH was characterized as described previously [47] by enzymatic activity. The enzymatic activity was determined as described previously [47] with 10 mM aqueous paraoxon solution at 405 nm using an Agilent 8453 UV-visible spectroscopy system (Agilent Technology, Waldbronn, Germany) equipped with a thermostated cell monitoring the accumulation of hydrolysis product, 4-nitrophenolate anion, at 25 °C and 405 nm (ε 17,000 M^−1^·cm^−1^, pH 9.0; ε 18,000 M^−1^·cm^−1^, pH 10.5). One unit of enzymatic activity (U) was defined as the quantity of the enzyme necessary to hydrolyze 1 μmol of paraoxon per min at 25 °C. The specific activity of the purified enzyme was 4340 U·protein·mg^−1^.

To obtain EPC, the PLE_50_ or PLD_50_ solution aliquot prepared in distilled water at a concentration of 20 mg·mL^−1^ was added to the solution of highly purified His_6_-OPH enzyme in 0.1 M phosphate buffer (pH 7.5) (protein concentration 0.16 ± 0.01 mg·mL^−1^, activity was 695 ± 15 U·mL^−1^). The aliquot volume was calculated so that the molar “enzyme:polymer” ratio was 1:5. Thereafter, the mixture was held for 30 min at +8 °C. The effective hydrodynamic diameter of particles of the prepared complexes was determined at 25 °C by DLS using a Zetasizer Nano ZS (Malvern Instruments Ltd., Malvern, UK) and was equal to 35 ± 5 nm.

### 2.3. Immobilization Technique Processing of Mineral Carrier with Non-Equilibrium Low Temperature Plasma

The original laboratory unit previously developed at the Moscow Institute of Physics and Technology and patented is equipped with a plasma generator [53], which provides the barrier discharge and non-equilibrium low temperature plasma, was used for the processing of the mineral carriers [43]. Several 1 g mineral samples were placed between the electrodes separated by a dielectric (quartz glass) and the NELTP generator was connected to the source. Then, in the zone for processing the raw material, an area of non-equilibrium low temperature plasma in a barrier discharge was produced; the voltage between the electrodes was up to 8000 V, frequency was up to 40 kHz [43]. The processing duration did not exceed 1.5 min. The reduced electric field value (E/N) was 15 × 10^−16^ V·cm^−2^, where E is electric field intensity, N is the total number of particles.

In order to obtain the immobilized enzyme preparation, 50 mL of the purified His_6_-OPH or the enzyme polyelectrolyte complex was mixed with 10 g of the mineral carrier and left at 8 °C for 6 h with periodic stirring [54]. Then the carrier was separated and washed with a solution containing 10 mM of NaHPO_4_ and 5 mM of NaHCO_3_ (pH 7.5) to constant residual activity of the immobilized enzyme. The specific activity of the produced chemicals was 231 ± 5 U/g of dry carrier for His_6_-OPH/PLE_50_ on zeolite and 236 ± 5 U/g of dry carrier for His_6_-OPH/PLD_50_ on zeolite. The dry weight of all the samples was determined according to the well-known method [55].

### 2.4. Preparation of Soil and Its Treatment with Enzymatic Preprationss

The development of the optimization technique was performed using sand soil, since positive results were previously obtained on fermentative decomposition of pesticides in soils of such type [42,43]. The treatment of the sand soil (initial humidity 90 ± 5%) for choosing the alkalizing agent was performed as follows: after introduction of the alkalizing agent (hydrated calcitic lime, dolomite powder, or birch ash) at a concentration of 10 g/kg soil the soil was stirred, and pH level was measured after 15 min. Soil pH was measured in a water suspension for each soil using a soil: solution ratio of 1:5 (pH_w_). When necessary, additional portions of the agent were added to achieve the pH level of 8.0–8.4. Then the solution of the enzymatic preparations of His_6_-OPH in 50 mM phosphate buffer (pH 8.0) with concentration of 300 U/kg soil was applied to the sample via surface sprinkling. The residual activity of the enzyme in the soil was determined in 1 h and 48 h [42] and the pH of the sample was measured. The samples were stored at +24 °C in the course of this study.

In the course of the studies for optimizing the chlorpyrifos destruction process, Dursban was diluted with distilled water and sprayed on the soil samples. The final concentration of pesticide in all the soil samples was 100 mg chlorpyrifos/kg soil. 48 h after introduction of the Dursban into the soil sample the enzymatic destruction of the pesticide was initiated.

The study for choosing the most efficient form of His_6_-OPH application for sandy soil proceeded as follows: first, the soil’s pH was increased up to 8.4 ± 0.03 using hydrated calcitic lime Ca(OH)_2_. Then NELTP-modified zeolite containing the immobilized His_6_-OPH-containing agent was added to the soil, or His_6_-OPH was sprayed on the soil either in its free form or as complexes with polyelectrolytes. The specific activity of the enzyme chemicals introduced into the soil was 300–1000 U/kg soil. Then the obtained substance was thoroughly mixed and exposed at room temperature for 48 h. After the 48-h-long exposure the residual concentration of chlorpyrifos in the analytical samples was evaluated.

In the course of the study of chlorpyrifos destruction in various soil types by enzyme polyelectrolyte complexes His_6_-OPH/PLE_50_ and His_6_-OPH/PLD_50_ immobilized on zeolite the soil humidity was controlled; when necessary, the samples were wetted to keep the humidity at the level of 80 ± 3%, and stirred. The soil’s humidity was evaluated with a TDR-100 professional humidity meter for soil and sand (Spectrum Technologies PLC, Bridgend, UK). Portions of the soil were taken at certain intervals and concentration of chlorpyrifos in them was analyzed.

### 2.5. Determination of Chlorpyrifos Decomposition in Soil Samples

In order to determine the concentrations of chlorpyrifos the pesticide was extracted from 3 g of soil with three portions of ethyl acetate (5 mL). The extracts were mixed, evaporated and dissolved in acetonitrile. Chlorpyrifos extracts were analyzed using HPLC (Knauer Smartline Pump 1000, Knauer Smartline UV Detector 2600, Berlin, Germany) and a Diasfer 110-C18 5 μm, 4.0 × 250 mm reverse-phase chromatography column (Biochemmack CT, Moscow, Russia) with a spectrophotometric detector (274 nm) and isocratic elution. Acetonitrile-water mixture (60:40) was applied as the eluent. The retention time for chlorpyrifos was 31 min. The eluent flow rate was 1 mL·min^−1^, and the detector cell temperature was 25 °C. The sample volume was 20 μL. 

The concentration of chlorpyrifos degraded by enzyme preparation was calculated by the formula Ce = Cc − Cd [42], where Ce is the actual concentration of pesticide degraded by enzyme in soil, Cc is the pesticide concentration in soil without immobilized or non-immobilized enzyme (control), Cd is the determined pesticide concentration in soil with immobilized enzyme preparation.

### 2.6. Assessment of Soil Samples Toxicity Using Photobacteria

Bioluminescent photobacteria have been successfully applied for soil ecotoxicity assessment [43,56]. Therefore, in this study it was decided to analyze the overall toxicity of the soil samples using photobacteria cells immobilized in poly (vinyl alcohol) (PVA) cryogel. We have previous experience in the successful use of such cells for investigating carriers containing organophosphorus compounds, in addition to the control of paraoxon residue concentrations during its enzymatic decomposition in soil samples [57].

*Photobacterium phosphoreum* photobacteria immobilized in PVA cryogel were prepared according to the previously described method [57]. To evaluate the toxicity of all the soil samples, the ecotoxicant was extracted from the soil samples at 22 ± 1 °C for 30 min on a thermostated shaker at a rate of 120 rpm using 2% aqueous solution of NaCl containing 3% (vol.) ethanol, using 5 mL of an extractant per 0.35 g of soil. The obtained extract samples were analyzed for toxicity in discrete mode using immobilized photobacteria. The bioluminescence of the immobilized photobacteria was analyzed using a LKB 1250 luminometer (LKB Wallac, Turku, Finland) and its level was expressed in mV. Bioluminescence detection was performed in aqueous 2% NaCl solution at 10 ± 1 °C. The initial level of cell luminescence was determined for 10 s at 10 °C after thermal equilibration of the system, and residual bioluminescence level was analyzed after 0.5 h upon exposure of immobilized photobacteria in the analyzed soil extract. The tests were performed in triplicate.

### 2.7. Statistical Data Processing

The data are presented as means of at least three independent experiments ± standard deviation (±SD). One-way ANOVA and Tukey test were applied. Statistical analysis was realized using SigmaPlot (ver. 12.5, Systat Software Inc., San Jose, CA, USA).

## 3. Results

### 3.1. The Choice of Conditions and Sequence of Operations to Increase the Efficiency of Applying His_6_-OPH-Based Enzyme Biocatalysts for Chlorpyrifos Degradation in Soil

His_6_-OPH functions efficiently at alkaline pH levels, while increasing the soil pH in excess of 8.5 can have a negative impact on the soil fertility. Therefore, hydrated calcitic lime, dolomite powder, and ash were tested for controlled increase of the soil pH up to 8.5 (Table 1). Sand soil with initial pH 7.4 ± 0.2% was used. Special attention was paid to the possibility of achieving the soil pH of 8.0–8.4 and keeping it in that range, as well as to the influence of the alkalizing agent on the stability of the enzymatic hydrolyzing activity towards organophosphorus pesticides.

The obtained data show (Table 1) that a smaller quantity of the agent was required to increase the soil pH to the target level when using hydrated calcitic lime than was the case with other alkalizing agents. Besides, the enzymatic activity in calcitic lime-containing soil remained higher during the 48 h upon introduction. It is also known from published data that introduction of Ca(OH)_2_ into the soil causes a decrease in bioaccessibility of heavy metals to the plant roots, and heavy metals can be present in the contaminated soils [58]. Thus, despite a slightly greater decrease of the soil’s pH in 48 h upon introduction of Ca(OH)_2_ (compared with other alkalizing agents studied), hydrated lime dust can be recommended as the best choice as pH controlling agent for assisting the use of His_6_-OPH-containing preparations for Chlorpyrifos destruction.

The following procedure was suggested based on the analysis of both the published data and our own experiments. This procedure ensured the highest efficiency of Chlorpyrifos hydrolysis in soils using His_6_-OPH-based agent. First, pH of the soil was measured; then it was increased up to 8.4 ± 0.03 via introduction of hydrated calcitic lime Ca(OH)_2_; the soil was wetted up to 60–80% (when the initial humidity was below 60%), the enzymatic agent was introduced, and the soil was thoroughly stirred to the depth of 10–30 cm.

### 3.2. Choice of the Form of the His_6_-OPH-Based Biocatalysts for Introduction into Soils for Destroying Chlorpyrifos

The possibility of decomposing 100 mg/kg soil of chlorpyrifos in model sandy soil was studied using various forms of enzymatic biocatalysts based on His_6_-OPH. In the course of this study we adhered to the sequence of operations and the set of conditions formulated above. In 48 h upon introduction of the enzymatic biocatalysts the residual concentration of chlorpyrifos in the samples was assessed (Table 2). 

The chlorpyrifos dose (100 mg/kg soil) introduced into the model sand soil was chosen based on the concentrations of the pesticide Dursban recommended for application in agriculture (0.8–3 L/ha) and the information that the non-decomposed chlorpyrifos can be accumulated in soil to reach concentrations as high as 100 mg/kg soil. The initial dose of the enzyme introduced into the soil was varied in the range of 300–1000 U/kg soil using the data available to the authors concerning the results of enzymatic hydrolysis of paraoxon in soils [43] and the relation of the enzymatic activity towards paraoxon and chlorpyrifos [46]. Samples of the initial soil contaminated with pesticide but containing no enzymatic preparations were used as control. All the samples were wetted as stirred in the same way.

The differences in the mean values among the groups 1–5 (Table 2) are greater than would be expected by chance according to one-way analysis of variance (ANOVA), there is a statistically significant difference (for 300 U/kg soil and 600 U/kg soil *p* = 0.010, for 1000 U/kg soil *p* = 0.008). According to Tukey test, the differences in the mean values of His_6_-OPH/PLD_50_ and His_6_-OPH/PLE_50_ (300–600 U/kg soil) so as His_6_-OPH/PLD_50_ and His_6_-OPH/PLE_50_ on zeolites (300–1000 U/kg soil) were not great enough to exclude the possibility that the difference is due to random sampling variability, there was not a statistically significant difference (*p* > 0.05). In other comparisons there were statistically significant differences (*p* < 0.05), so the residual concentration of chlorpyrifos seems to be dependent of the form of the enzymatic biocatalysts. 

Without considering the economic factors, the best dosage for destruction of chlorpyrifos in soil is 1000 U/kg soil, because it allows achieving 100% destruction of the pesticide within 48 h when polyelectrolyte complexes of His_6_-OPH are used. Thus, since the specific activity of the initial agents for His_6_-OPH/PLE_50_ immobilized on zeolite was 231 ± 5 U/g of dry carrier and that of similarly immobilized His_6_-OPH/PLD_50_ was 236 ± 5 U/g of dry carrier, introduction of 4 g of zeolite containing immobilized enzyme per kg of soil can be recommended for complete destruction of chlorpyrifos in soil. This is well within the norms recommended for the use of zeolites in agriculture, namely, up to 10–20 volume % of the soil.

### 3.3. The Chlorpyrifos Hydrolysis in Different Types of Soil

The biocatalysts based on the natural zeolite processed with the NELTP for 1.5 min, and the enzyme polyelectrolyte complexes His_6_-OPH/PLE_50_ and His_6_-OPH/PLD_50,_ showed the best overall performance in terms of catalytic activity and stability against elution [43]. Therefore, they can be recommended for use in the agricultural technologies for the OPC decomposition. The poly-l-glutamic acid is more affected by hydrolytic enzymes than the poly-l-aspartic acid [59]. Therefore, the His_6_-OPH/PLE_50_ biocatalyst was selected for the experiments on OPC decomposition in soils. It was argued that during the practical use such biocatalysts will undergo biodegradation easier under natural conditions upon completion of their primary function, i.e., hydrolysis of the OPC.

Using the suggested approach the kinetics of chlorpyrifos destruction in soils of various types was studied. Regular taking of samples was performed in the course of the experiment, and the residual chlorpyrifos content was evaluated, as well as enzymatic activity and soil humidity. When the humidity of the soil dropped below 50%, the sample was additionally wetted by spraying.

**Figure 1:**
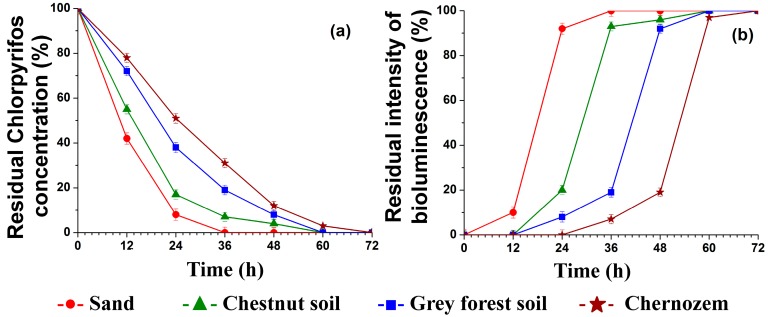
Destruction of 100 mg·kg^−1^ soil of chlorpyrifos under the action of His_6_-OPH/PLE_50_ immobilized in the plasma-modified (for 1.5 min) natural zeolite in various types of soil (**a**) and residual bioluminescence of immobilized *Photobacterium phosphoreum* used for evaluating the residual toxicity of the treated soil samples (**b**).

It was found (see Figure 1a) that upon introducing the biocatalyst in a dose of 1000 U/kg into soils of different composition, the chlorpyrifos hydrolysis successfully occurred as early as during the first day (49–92%). The complete decomposition was achieved within 72 h even when using the extremely high concentration of chlorpyrifos (100 mg·kg^−1^ soil).

When using photobacteria cells immobilized in the PVA cryogel, a toxicity assessment of the aqueous extracts made from the soil samples was conducted before and after the use of the His_6_-OPH/PLE_50_-based biocatalyst. The results (Figure 1b) showed a complete absence of toxicity in soil samples within 72 h after the treatment with the enzyme biocatalyst. Similar results were obtained when testing (under the same conditions) the biocatalyst based on the natural zeolite processed with the NELTP for 1.5 min and the His_6_-OPH/PLD_50_ polyelectrolyte complex containing poly-l-aspartic acid.

The results obtained for chlorpyrifos destruction by His_6_-OPH-based biocatalysts show that the hydrolysis occurs at a greater rate in soils with a relatively poor content of humic components (Figure 1a); these results are on the whole comparable with those obtained in the previous studies [42,43]. However, despite the differing rates of the hydrolysis process, the use of His_6_-OPH-based biocatalysts in accordance with the suggested technique allows achieving 100% destruction of chlorpyrifos in soils of various types.

## 4. Discussion

The choice and the efficiency of the various techniques for purification of pesticides from soil essentially depends on the type of the soil and its physico-chemical characteristics: humidity, pH, water- and air-permeability, etc. This was taken into account during the study for optimizing the techniques of applying enzymatic biocatalysts for destruction of chlorpyrifos (the basic component of the commercial chemical Dursban). Special attention was therefore paid to parameters such as soil humidity and pH, the level of pesticide destruction in the soil depending on stirring, etc.

Soil humidity influences the mobility of the pollutant and the structure of soil agglomerates. Water is also essential for the efficiency of biodegradation processes in soils, including those involving enzymatic biocatalysts. Thus, in order to optimize the conditions of chlorpyrifos hydrolysis catalyzed by His_6_-OPH, the humidity of soil should be controlled and kept in the range of 60–80%, which also facilitates plant growth [60]. Therefore, the technique for chlorpyrifos destruction with His_6_-OPH-based agents in soils with humidity below 60% should include at least one wetting of the soil (e.g., by sprinkling) immediately before, during, or just after the introduction of the enzyme.

It is known that upon treatment of agricultural plants with chlorpyrifos the greater part of the pesticide is localized in the surface layer of the soil at the depth less than 30–40 cm. Upon application of chlorpyrifos at 1.12 kg/ha the detected pesticide concentrations are 10 ppm at 7.5 cm below the surface, 0.3 ppm at 22.5 cm, and 0.01 ppm at the depth of 37.5 cm [61]. Thus, in order to optimize the catalysis process, the necessary condition for efficient chlorpyrifos destruction is soil stirring to the depth of 10–30 cm. Standard agricultural appliances can be used for this purpose in the field conditions.

It is also known that organophosphorus pesticides are more efficiently hydrolyzed in an alkaline medium. Thus, an increase of soil pH from 6 to 8 causes an essential increase in the chlorpyrifos degradation rate (by a factor of 2–3) [12]. Besides, the optimal pH for His_6_-OPH activity is 10.5, and the effective working range is 7.5–11.5, which is in the alkaline domain. Therefore, in the course of pesticide hydrolysis by His_6_-OPH the main attention should be paid to maintaining control of the soil pH and keeping it above 7.0. Some of the soil types in the subtropical belt (e.g., humus-carbonate, carbonate alluvial, and marl soil) used for agricultural purposes where chlorpyrifos-based chemicals are currently applied are themselves slightly alkaline (pH 8.0–8.4). The pH of acidic soils can be regulated by introducing the well-known and inexpensive substances extensively used for alkalizing, such as lime kiln, dolomite powder, ash, etc. However, to preserve the soil’s fertility, its pH should be kept below 8.5. In the course of optimizing the enzymatic purification of soils it is important to choose such substances for controlling the soil’s pH which would ensure stable functioning of the enzymatic biocatalysts in terms of catalytic action with respect to chlorpyrifos hydrolysis. Thus, before introducing His_6_-OPH-based chemicals into the soil the pH of the latter should be checked. If pH is below 8.0, a substance routinely used in agriculture for soil alkalizing should be added to keep pH in the range of 8.0–8.4. In our study the best results were achieved for Ca(OH)_2_ (Table 1).

In the course of optimizing the techniques for soil purification from pesticides using enzyme-based biological methods the choice of the form of the biochemical agent which would be introduced into the soil is also important, because the agent’s performance can be affected both by its form and by the environmental conditions. Two techniques were suggested and realized in the course of the experiments in order to stabilize the enzymatic activity, namely, inclusion of the enzyme into stable polyelectrolyte complexes based on polyaminoacids, and immobilization of these complexes on a mineral carrier, natural zeolite. It is important that all the components for the suggested technique are absolutely non-toxic towards soil and water biocenoses. The suggested methods are on the one hand based on the results obtained earlier and, on the other hand, thoroughly studied in the course of the current research on chlorpyrifos destruction in soils.

The use of enzymes with OPH activity in the immobilized form, in particular when adsorbed on natural cellulose-containing carriers [42,62], ensures a lasting high concentration of biocatalyst directly in the OPC localization. This technique also helps stabilizing the catalytic activity of the enzymatic biocatalyst at lower pH resulting from the accumulation of the ions H^+^ released during the hydrolysis of the OPC.

The His_6_-OPH-containing straw-based immobilized enzymatic preparations developed earlier ensured decomposition of 630 mg paraoxon or 850 mg diazinon or 185 mg parathion per kg soil in less than 10 days. The half-life of active biocatalyst in sand was estimated as 130 days [42]. However, the main drawback of these biocatalysts based on immobilized His_6_-OPH was their relatively low specific enzymatic activity per unit of the carrier, which was below 72 U/g of carrier. Enzyme interaction with cellulose-containing carriers was clearly caused by the capillary-porous structure of hygroscopic cellulose-containing material capable of retaining the enzyme solution within the macropores (in the lumens of plant cells–free moisture) or micropores (between the fibrils of the cytoderm–bound, or hygroscopic, moisture). 

It is known that when applied to the soil in order to improve its structure, the zeolites are a source of natural mineral supplement that can significantly improve crop yields, help soil aeration and retain water in the root zone [63]. Low cost, high prevalence in nature, and usability as sorbents make such minerals as zeolites and perlites promising carriers for the enzyme, microbial biomass and organic matter (humic acids) immobilization and their use in agricultural technologies [64,65]. However, mineral carriers are characterized by relatively low protein adsorption capacity compared to cellulose-containing ones and do not result in effective enzymatic biocatalysts, including those based on organophosphorus hydrolase [42]. Therefore, the specific activity of biocatalysts produced using various mineral carriers (vermiculite, activated carbon, Sibunit, sand, diatomaceous earth) did not exceed 5 U/g of carrier, which is 14.4 times lower than that of the straw-based ones.

The non-equilibrium low temperature plasma (NELTP) processing in barrier discharge can positively affect the sorption characteristics of mineral carriers applied as building materials [66,67]. In general, plasma is an ionized gas that can be generated by a number of methods, including electric discharges (glow, microwave, plasma jet, radio frequency, etc.). Depending on their energy level, temperature, and ionic density, plasmas are usually classified as high temperature ones (for nuclear applications) and low temperature ones (including thermal and cold plasmas). The bulk temperature in cold plasmas can be as low as room temperature [68].

When applying the enzymatic polyelectrolyte complex containing the parent enzyme His_6_-OPH and poly-l-glutamic acid (PLE_50_), immobilized on the plasma-modified (for 1.5 min) natural zeolite, in a dose of 300 U/kg soil at the initial extremely high concentration of paraoxon of 650 mg·kg^−1^ soil, complete lack of residual toxicity was achieved in a rather short time (after 180 h) in all the tested soil samples [43]. The performed enzyme surface modification with polymers (due to the interaction of oppositely charged molecules of the enzyme and the polymer) resulted in the formation of complexes with high enzymatic activity and increased stability of the His_6_-OPH. The use of polyanions when developing the enzyme polyelectrolyte complexes apparently does not directly affect the active center of the enzyme and has a positive effect on the preservation of the His_6_-OPH activity.

In the current study for optimization of His_6_-OPH application technique for chlorpyrifos destruction in soils the best results were also obtained for hydrolase in the form of polyelectrolyte complexes immobilized on zeolite (Table 2), which agrees well with the previously obtained data on paraoxon hydrolysis in soils [43]. Note that better results on neutralization of organophosphorus compounds in soils using His_6_-OPH-based agents were achieved in the current study, which is most probably due to the suggested approaches including the increasing of the soil pH to 8.0–8.5 using special alkalizing agents and keeping the soil humidity above 60%.

The results obtained show that application of the suggested optimized approach to chlorpyrifos destruction using His_6_-OPH-based enzymatic biocatalysts ensures more rapid pesticide neutralization than in case of using known microbial agents for this end. Thus, microbial biocatalyst for degradation of organophosphorus pesticides based on bacterial cells of *Stenotrophomonas* YC-1 strain was used in study [69] for detoxification of soil polluted with chlorpyrifos (100 mg/kg). This approach ensured a high enough initial chlorpyrifos degradation rate (19 mg/kg_soil_/day), but this rate was much lower than when using His_6_-OPH in soils of various types (49–92 mg/kg_soil_/day) (Figure 1a).

The possibility of microbial detoxification of soil polluted with chlorpyrifos at 50 mg/kg_soil_ was shown in [70]. Bacterial cells of the species *Pseudomonas aeruginosa*, *Bacillus cereus*, *Klebsiella* sp., *Serratia marscecens* were used as biological agents to destroy the pesticide. *P. aeruginosa* cells had the highest efficiency in bioremediation of the polluted soil. Other microorganisms studied caused slower degradation of chlorpyrifos. The main drawback of the suggested microbial remediation technique is that these bacteria are pathogens which can cause pneumonia, sepsis, infections of intestine and urogenital system, wound infections etc. with possible lethal consequences [71,72]. Another disadvantage of this technique is the great duration of the soil purification process, which limits the practical applicability of this method. This approach also requires introduction of additional carbon and nitrogen sources into the treated soil to facilitate the growth of the pesticide-decomposing microorganisms, which increases the total cost of this technique.

The most practically important result of the present study is the observation that the His_6_-OPH-based biocatalysts can efficiently destroy chlorpyrifos in soils with poor humus content, where application of the traditional techniques involving live microbial consortia is limited. Thus the growing demand is observed in 21st century for the products of palm agriculture: palm and coconut oil, bananas, and dates. Agricultural palm plantations involve still newer regions with tropical and subtropical climate. The palm agriculture takes lead compared with other plant cultivation spheres in such countries as Pakistan [13,73]. Most of the soil resources in the subtropics are poor in humus content, lack structure, and are heavy in mechanical terms. The subtropical climate is nonetheless favorable for efficient growing of many useful agricultural products. Such territories, apart from palms, yield citrus fruit, persimmon, figs, pomegranates, olives, laurel, avocadoes, walnuts, almonds, pistachios, peaches, apples, and other fruit. Chlorpyrifos is often used in agriculture, and can be accumulated in soil, water, and agricultural produce. The strategy for enzymatic purification of soils from pesticides is perspective for bioremediation of such soils.

## 5. Conclusions

The optimized conditions and sequence of operations were found for using His_6_-OPH for destruction of chlorpyrifos in soils of various types. First stage was increasing the soil pH to 8.4 using hydrated calcitic lime Ca(OH)_2_. The second one is introduction into the soil of the enzyme preparation at 1000 U/kg soil (corresponding to 4 g zeolite/kg soil) in the form of NELTP-modified zeolite with immobilized relatively inexpensive polyelectrolyte complexes containing the enzyme His_6_-OPH and polyanionic polymer: poly-l-glutamic acid (PLE_50_) or poly-l-aspartic acid (PLD_50_). The third one is wetting the soil to achieve 60–80% humidity, followed by through stirring of the soil to the depth of 10–30 cm and exposure for 48–72 h. In case of initial chlorpyrifos concentration of 100 mg/kg soil complete destruction of the pesticide is achieved within 72 h when using the optimized technique of enzymatic treatment.

Compared to the existing biological techniques for purification of soils from pesticides based on the use of live organisms, using His_6_-OPH-based enzymatic biocatalysts was found to be preferable for humus-poor soils, such is sand and loam-sand chestnut soils, as well as those with alkaline pH (8.0–8.4), located in desert-steppe, foothill, and subtropical territories where chlorpyrifos is still actively used.

The suggested approach is based on technological operations which can be reproduced under the actual agricultural and farming conditions using standard equipment. This approach ensures the decrease of environmental risks and is aimed at preserving health of humans and animals, which promises the high market potential of the developed techniques. The economic feasibility of the suggested method is also based on the low cost of the fermentation agent, because the latter can be produced in quantity due to the use of highly productive bacterial strain with high yield of the His6-OPH ferment [51].

## Figures and Tables

**Table 1:** The choice of pH controlling agent in the course of choosing the scheme of applying His_6_-OPH for Chlorpyrifos destruction in soil.

pH Controlling Agent	Dose Introduced into Soil (g/kg soil)	Soil pH after Introduction of Agent		Residual Activity of the Enzyme upon Introduction into the Soil (%)	
		In 15 min	In 48 h	In 1 h	In 48 h
Control (no agent)	-	7.4 ± 0.02	7.4 ± 0.03	84 ± 2	51 ± 2
Hydrated calcitic lime	22 ± 1	8.4 ± 0.03	8.0 ± 0.03	93 ± 2	60 ± 2
Dolomite powder	64 ± 1	8.2 ± 0.05	8.1 ± 0.03	91 ± 2	50 ± 2
Birch ash	120 ± 3	8.1 ± 0.02	8.0 ± 0.02	91 ± 2	45 ± 2

**Table 2:** Residual concentration of chlorpyrifos (%) in sand soil in 48 h upon introduction of the enzymatic biocatalysts into the soil *.

Group	Form of the Enzymatic Biocatalysts	Initial Dose		
		300 U/kg soil	600 U/kg soil	1000 U/kg soil
1	His_6_-OPH	74 ± 4	61 ± 3	48 ± 3
2	His_6_-OPH/PLD_50_	48 ± 2	27 ± 1	13 ± 0.5
3	His_6_-OPH/PLE_50_	43 ± 2	29 ± 1	8 ± 0.3
4	His_6_-OPH/PLD_50_ on zeolite	27 ± 1	19 ± 0.5	0
5	His_6_-OPH/PLE_50_ on zeolite	29 ± 1	17 ± 0.5	0

* Residual concentration of chlorpyrifos in soil after 48 h without introduction of the enzymatic biocatalysts was 87 ± 1%.
